# Herpetrione, a New Type of PPARα Ligand as a Therapeutic Strategy Against Nonalcoholic Steatohepatitis

**DOI:** 10.34133/research.0276

**Published:** 2023-11-30

**Authors:** Lang Linghu, Wei Zong, Yixuan Liao, Qianyu Chen, Fancheng Meng, Guowei Wang, Zhihua Liao, Xiaozhong Lan, Min Chen

**Affiliations:** ^1^ Key Laboratory of Luminescence Analysis and Molecular Sensing (Southwest University), Ministry of Education, College of Pharmaceutical Sciences, Southwest University, Chongqing 400715, China.; ^2^ School of Pharmacy, Zunyi Medical University, Zunyi 563000, China.; ^3^ School of Life Sciences, Integrative Science Center of Germplasm Creation in Western China (CHONGQING) Science City and Southwest University, TAAHC SWU Medicinal Plant Joint R&D Centre, Southwest University, Chongqing 400715, China.; ^4^ TAAHC-SWU Medicinal Plant R&D Center, Tibet Agricultural and Animal Husbandry University, Nyingchi, Tibet 860000, China.

## Abstract

Non-alcoholic fatty liver disease, especially nonalcoholic steatohepatitis (NASH), is a leading cause of cirrhosis and liver cancer worldwide; nevertheless, there are no Food and Drug Administration-approved drugs for treating NASH until now. Peroxisome proliferator-activated receptor alpha (PPARα) is an interesting therapeutic target for treating metabolic disorders in the clinic, including NASH. Herpetrione, a natural lignan compound isolated from Tibetan medicine *Herpetospermum caudigerum*, exerts various hepatoprotective effects, but its efficacy and molecular mechanism in treating NASH have not yet been elucidated. Here, we discovered that herpetrione lessened lipid accumulation and inflammation in hepatocytes stimulated with oleic acid and lipopolysaccharide, and effectively alleviated NASH caused by a high-fat diet or methionine-choline-deficient diet by regulating glucolipid metabolism, insulin resistance, and inflammation. Mechanistically, RNA-sequencing analyses further showed that herpetrione activated PPAR signaling, which was validated by protein expression. Furthermore, the analysis of molecular interactions illustrated that herpetrione bound directly to the PPARα protein, with binding sites extending to the Arm III domain. PPARα deficiency also abrogated the protective effects of herpetrione against NASH, suggesting that herpetrione protects against hepatic steatosis and inflammation by activation of PPARα signaling, thereby alleviating NASH. Our findings shed light on the efficacy of a natural product for treating NASH, as well as the broader prospects for NASH treatment by targeting PPARα.

## Introduction

Non-alcoholic fatty liver disease (NAFLD) is one of the most widespread chronic liver conditions globally, accounting for 30.05% of all cases [1]. It is defined by benign lipid accumulation and comprises a wide range of clinical conditions, including hepatic steatosis and nonalcoholic steatohepatitis (NASH), ultimately progressing to fibrosis, cirrhosis, and liver cancer [2]. NASH represents a severe form of NAFLD typified by hepatic injury, featuring hepatocyte ballooning, inflammatory cell infiltration, and progressive fibrosis [3]. When NASH develops, pharmacological intervention is essential to keep the disease from progressing to more severe stages. Nonetheless, the Food and Drug Administration has yet to approve any medicine for effectively treating NASH [4].

Nuclear receptors are transcription factors activated by ligands, which are crucial in inflammatory and metabolic diseases, such as pancreatitis and NASH [5,6]. Peroxisome proliferator-activated receptors (PPARs) are a kind of transcription factors that include PPARα, PPARβ, and PPARγ, each of which has a distinct tissue distribution and functions [7–9]. PPARs have been recognized as critical regulators of inflammation and fatty acid oxidation, making them appealing targets for treating NASH [10,11]. PPARα, one of them, is expressed heavily in the liver and is currently being investigated as a therapeutic target for metabolic disorders like NASH [12]. Previous reports have suggested that PPARα activation is beneficial for improving steatosis, inflammation, and fibrosis in preclinical models of NASH. Treatment with a PPARα agonist has been shown to protect wild-type mice from steatohepatitis and steatosis when given a methionine-choline-deficient (MCD) diet [13]. PPARα deficiency, nevertheless, results in more severe steatohepatitis in MCD diet mice, providing additional evidence of PPARα involvement in NASH [14]. Fibrates, acting as synthetic PPARα agonists, diminish triglyceride (TG) and low-density lipoprotein cholesterol (LDL-c) content while increasing high-density lipoprotein cholesterol (HDL-c) content. Consequently, they have been recommended as lipid-lowering drugs to treat dyslipidemia in the clinic [15,16]. Of note, there is a decrease in PPARα expression as liver fibrosis progresses in NASH patients [9]. Therefore, the development of novel PPARα agonists that exhibit greater potency and efficacy could provide more utility in NASH treatment. These evidences suggest that PPARα has potential in the therapeutic field of NASH.

Herpetrione is a lignan component isolated from *Herpetospermum caudigerum*, which has long been used to remedy liver diseases, cholic diseases, and dyspepsia; it is found primarily in Tibet, northeast Nepal, and India [17,18]. Lignans from *H. caudigerum* show beneficial effects against hepatobiliary disease [19,20]. Herpetrione had a prominent inhibitory effect on HBV DNA according to previous research [21]. Herpetrione also protects against D-Gal- and CCl_4_-induced liver injury by lowering plasma alanine aminotransferase (ALT) and aspartate aminotransferase (AST) content and improving histopathological damage [22,23]. However, the efficacy of herpetrione on NASH is not yet explicit.

As a result, the present study sought to research the efficacy of herpetrione on NASH and the involvement of PPARα signaling in herpetrione-induced NASH modulation. The findings revealed that herpetrione reduces hepatic lipid accumulation and inflammation. Particularly, via its direct connection to PPARα, herpetrione considerably hinders the progress of NASH, shedding light on its efficacy as a natural product for the treatment of NASH.

## Results

### Herpetrione attenuates cellular lipid accumulation and inflammation in HepG2 cells stimulated with OA and LPS

**Fig. 1.**
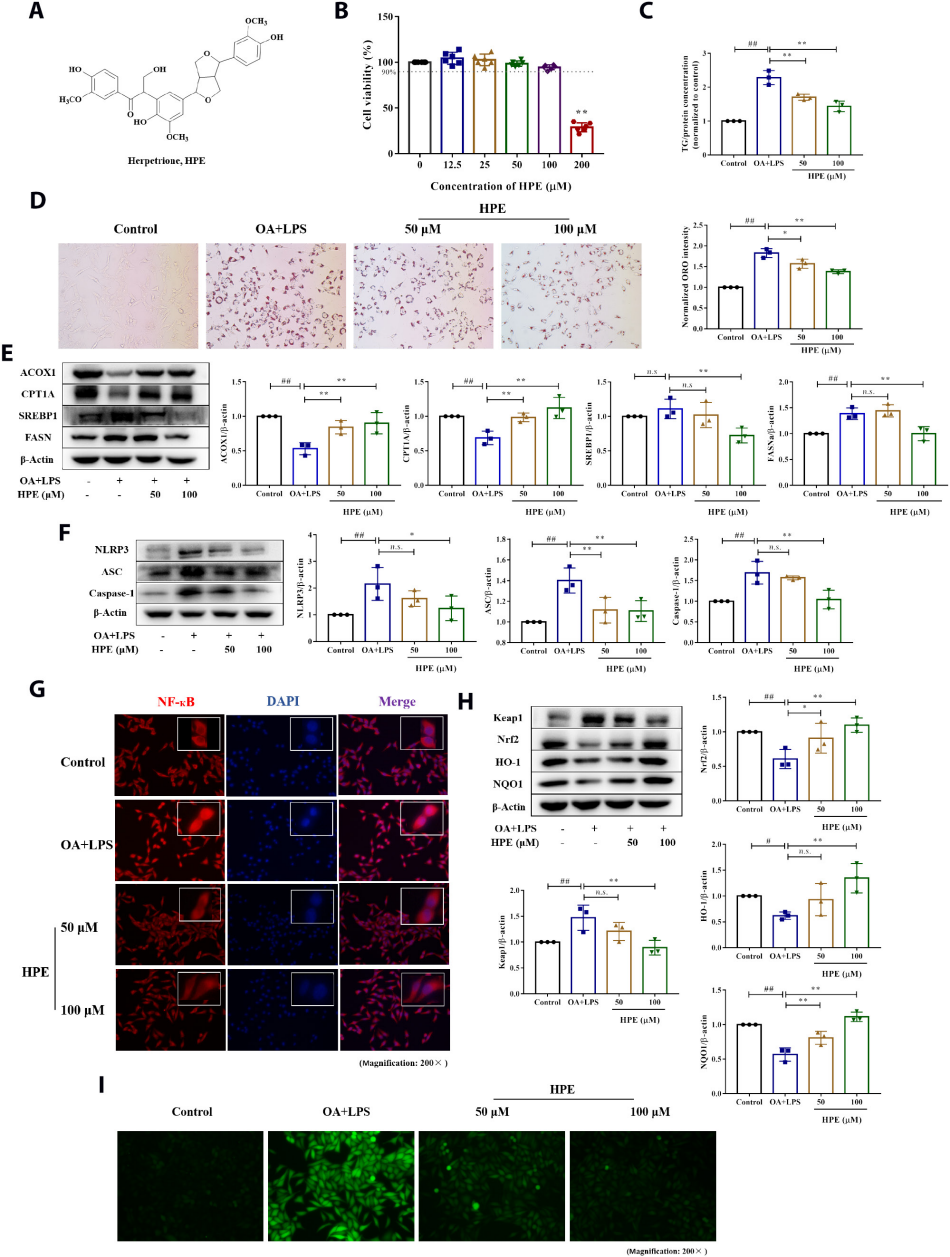
Herpetrione reduces lipid accumulation and inflammation in HepG2 cells stimulated with OA and LPS. (A) Chemical structure of herpetrione. (B) Cytotoxicity of herpetrione in HepG2 cells. (C) TG content detection of HepG2 cells treated with herpetrione (50 and 100 μM) in response to OA and LPS for 24 h. (D) Representative ORO staining images of HepG2 cells in the indicated groups. The magnification is set to 200×. (E) Representative Western blot of lipid metabolism-related molecules (ACOX1, CPT1A, SREBP1, and FASN) in HepG2 cells. (F) Representative Western blot of inflammation-related molecules (NLRP3, ASC, and Caspase-1) in HepG2 cells. (G) NF-κB nuclear ectopic in HepG2 cells. (H) Protein expression of oxidative stress-related molecules (Keap1, Nrf2, HO-1, and NQO1). (I) Active oxygen content in HepG2 cells. *n* = 3 per group. β-Actin served as a loading control.

A model of cellular fat overaccumulation was enacted in hepatocytes by incubation with oleic acid (OA) and lipopolysaccharide (LPS) for 24 h in vitro. Herpetrione belongs to the class of lignans and is derived from *H. caudigerum*, was shown in Fig. 1A. The results of methyl thiazolyl tetrazolium assay revealed that herpetrione was non-toxic on HepG2 cells at concentrations under 100 μM (Fig. 1B), in which 50 and 100 μM were used to the following experiments. The lipid-lowering efficacy of herpetrione was verified by detecting TG content in HepG2 cells (Fig. 1C); then, herpetrione obviously reduced cellular lipid droplet accumulation, according to Oil Red O (ORO) staining (Fig. 1D). Furthermore, in OA- and LPS-treated hepatocytes, herpetrione treatment increased the protein levels of the lipid oxidation molecules acyl-CoA oxidase 1 (ACOX1) and carnitine palmitoyl transferase 1A (CPT1A) while decreasing the levels of the lipogenic proteins sterol regulatory element-binding protein 1 (SREBP1) and fatty acid synthase (FASN) (Fig. 1E). Semblable results were demonstrated in AML12 cells (Fig. S1). Additionally, the expression of NLRP3 inflammasome components was reduced by herpetrione in HepG2 cells cultured in OA- and LPS-containing medium (Fig. 1F). Nuclear migration of the nuclear factor NF-кB is required for NLRP3 inflammasome activation. Treatment with herpetrione inhibited NF-кB entry into the nucleus (Fig. 1G). Moreover, steatosis additionally triggers cytotoxic events such as oxidative stress, which may result in liver damage. Herpetrione treatment decreased the emergence of intracellular reactive oxygen species, as well as regulate protein expression involved in oxidative stress pathways (Fig. 1H and I). These findings suggest that herpetrione attenuates steatohepatitis-related alterations and inflammation in hepatocytes.

### Herpetrione ameliorates hepatic steatosis and insulin resistance in high-fat diet-fed mice

**Fig. 2.**
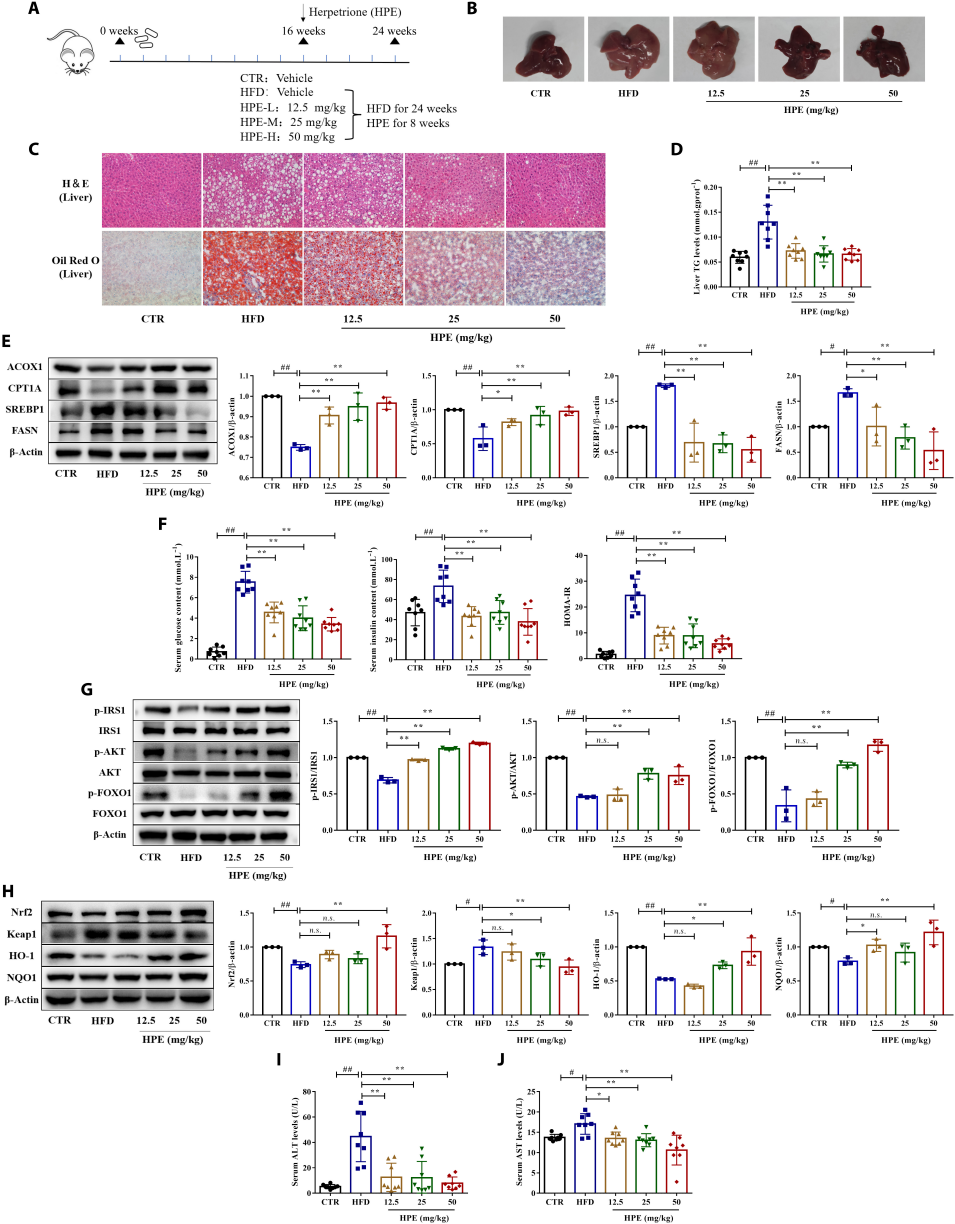
Herpetrione mitigates HFD-induced hepatic steatosis and insulin resistance in mice. (A) The experimental procedure to examine the effect of herpetrione in HFD-induced NAFLD in mice is outlined in the diagram, with *n* = 8 per group. (B) Illustrative images of liver morphology. (C) Liver sections from the tested mice are shown in H&E and ORO staining images. The magnification is 200×. (D) Hepatic TG contents of the mice in the various groups. (E) Protein expression of lipid metabolism-associated molecules (ACOX1, CPT1A, SREBP1, and FASN) in mice. (F) Serum glucose, serum insulin, and HOMA-IR were measured in the specified mouse groups. (G) Expression of proteins associated with the insulin signaling pathway, including IRS1, AKT, and FOXO1, in mice. (H) Representative Western blot of oxidative stress-related molecules (Keap1, Nrf2, HO-1, and NQO1) in the indicated mice. (I and J) Serum concentrations of ALT (I) and AST (J) in mice were measured. Results are presented as mean ± SD, with β-actin serving as a loading control.

We further explored whether herpetrione could attenuate hepatic steatosis and insulin resistance in high-fat diet (HFD)-fed mice. Mice were fed an HFD for 16 weeks and follow administered herpetrione (12.5, 25, and 50 mg/kg), while continuing to obtain an HFD for an appended 8 weeks (Fig. 2A). Morphological analysis of the liver showed that HFD feeding resulted in a whitish liver surface and an increase in liver volume, whereas the liver had a ruddy surface and decreased volume after herpetrione treatment (Fig. 2B). Regarding white adipose tissue, herpetrione reduced the volume of adipocytes and decreased white adipose weight and the tissue index (Fig. S2A, B, and F). Compared to the control group, the weight of HFD-fed mice showed a trend of continuous increase; however, 50 mg/kg herpetrione reversed the weight gain (Fig. S2C). In HFD-fed mice, hematoxylin and eosin (H&E) and ORO staining revealed hepatocyte damage and steatosis; however, herpetrione treatment improved hepatic steatosis, ballooning, and lipid droplet formation in liver tissues (Fig. 2C). Long-term intake of an HFD seriously affected the ability of the liver to remove TG and lipids accumulated in the liver. Herpetrione significantly reduced serum LDL-_C_, liver TG, and total cholesterol (TC) levels (Fig. 2D and Fig. S2D and E). Excessive lipid synthesis or fatty acid oxidation impairment are the underlying mechanisms of steatosis. The upregulation of fatty acid oxidation-related proteins (CPT1A and ACOX1) and the downregulation of lipid synthesis-related proteins (FASN and SREBP1) were observed after treatment with herpetrione (Fig. 2E). In addition, the HFD enlargement in blood glucose and insulin content were reversed by herpetrione administration; simultaneously, herpetrione decreased the homeostasis model assessment of insulin resistance (HOMA-IR) index (Fig. 2F). The key mechanism of insulin resistance is dysfunction of the insulin signaling, while the IRS1/AKT/FOXO1 signaling pathway plays a vital role. According to our results, herpetrione increased the ratios of p-IRS1/IRS1, p-AKT/AKT, and p-FOXO1/FOXO1, which are markers associated with the IRS1/AKT/FOXO1 signaling pathway (Fig. 2G). Furthermore, the HFD-induced abnormalities in the protein level of Nrf2/Keap1 signaling pathway components were reversed after herpetrione treatment (Fig. 2H). The content of plasma ALT and AST was markedly reduced in the herpetrione-treated mice than the HFD group (Fig. 2I and J). Herpetrione therapy protects mice from obesity, insulin resistance, oxidative stress, and hepatic dysfunction caused by HFD, according to our findings.

### Herpetrione alleviates hepatic steatosis and inflammation in MCD-fed mice.

**Fig. 3.**
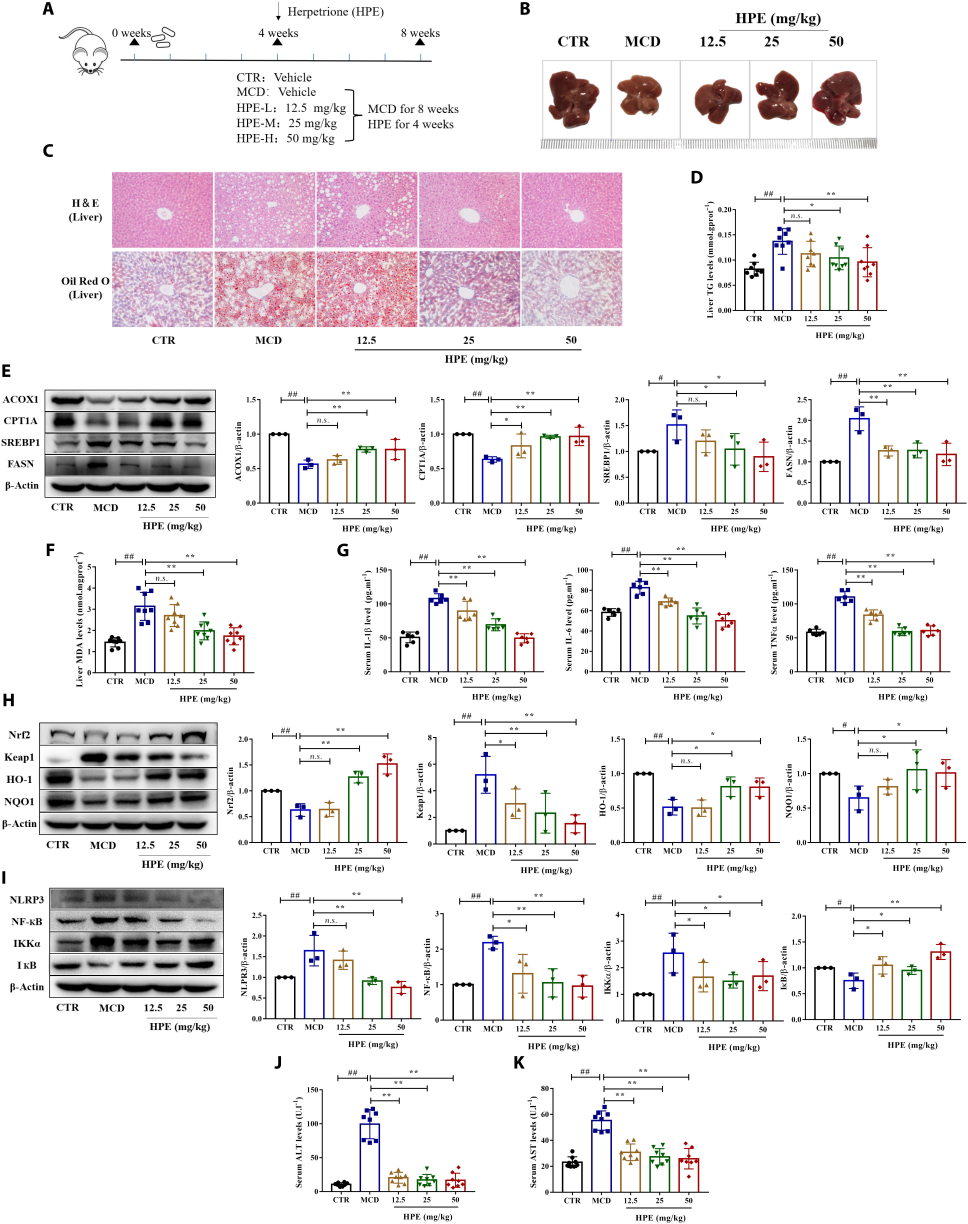
Herpetrione alleviates MCD-induced hepatic steatosis and inflammation in mice. (A) Schematic of the experimental methodology to evaluate the part played by herpetrione in MCD-induced NASH in mice, with a sample size of 8 per group. (B) Representative images of liver morphology. (C) H&E and ORO staining images of liver sections taken from the mice specified are presented. The magnification is set to 200×. (D) Hepatic TG levels of the mice in the specified groups. (E) The level of lipid metabolism-related protein (ACOX1, CPT1A, SREBP1, and FASN) in mice. (F) Liver oxidative stress marker MDA contents in the indicated group. (G) Serum inflammatory cytokine IL-1β, IL-6, and TNF-α contents in the indicated group. (H) Representative Western blot of oxidative stress-related molecules (Keap1, Nrf2, HO-1, and NQO1) in MCD-induced mice. (I) Protein expression of inflammation associated molecules (NLRP3, NF-κB, IKKα, and IκB) in mice. (J and K) Serum concentrations of ALT (J) and AST (K) were measured in the mice as indicated. β-Actin was used as a loading control. Results are presented as mean ± SD.

The MCD diet is commonly used in animal studies of NASH due to its high sucrose and fat content, as well as its lack of methionine and choline. An MCD-induced NASH model, which exhibits a more prominent inflammatory response and fibrosis than an HFD-induced animal, was employed to further study the effectiveness of herpetrione against NASH [24]. Mice were fed an MCD for 4 weeks, and then intervened with herpetrione while still receiving an MCD for another 4 weeks (Fig. 3A). Herpetrione treatment somewhat increased liver weight and the liver index (Fig. S3A to C) in MCD-fed mice. As expected, MCD-induced severe steatosis, inflammatory cell infiltration, and liver fibrosis were reversed by treatment with herpetrione (Fig. 3B and C and Fig. S3F). The liver TG level was reduced in mice treated with herpetrione (Fig. 3D), but the TC content was unaffected (Fig. S3D). Subsequent Western blot analysis also confirmed that herpetrione could ameliorate hepatic steatosis (Fig. 3E). Notably, herpetrione treatment had a marked superiority on the elevation of HDL-c levels in MCD-fed mice (Fig. S3E).

We measured the amounts of lipid peroxidation products because oxidative stress contributes to the onset and progression of hepatic injury in NASH. Malondialdehyde (MDA) is utilized as an indicator of lipid peroxidation, denoting the degree of cell membrane oxidation. MDA concentrations in mice were lowered after herpetrione treatment, and MCD-induced alterations in protein levels of Nrf2/Keap1 signaling pathway components were reversed (Fig. 3F and H). Furthermore, MCD-fed mice had a more severe inflammatory response, so we looked into whether herpetrione treatment could help with inflammation as well. Herpetrione therapy lowered inflammatory cytokine release and relieved inflammation via the NF-кB/NLRP3 pathway (Fig. 3G and I). ALT and AST levels in the plasma are major indicators of liver injury and were higher in an MCD mice than in the control group; however, herpetrione treatment lowered ALT and AST levels (Fig. 3J and K).

### Herpetrione promotes activation of PPARα signaling in HFD- or MCD-fed mice

**Fig. 4.**
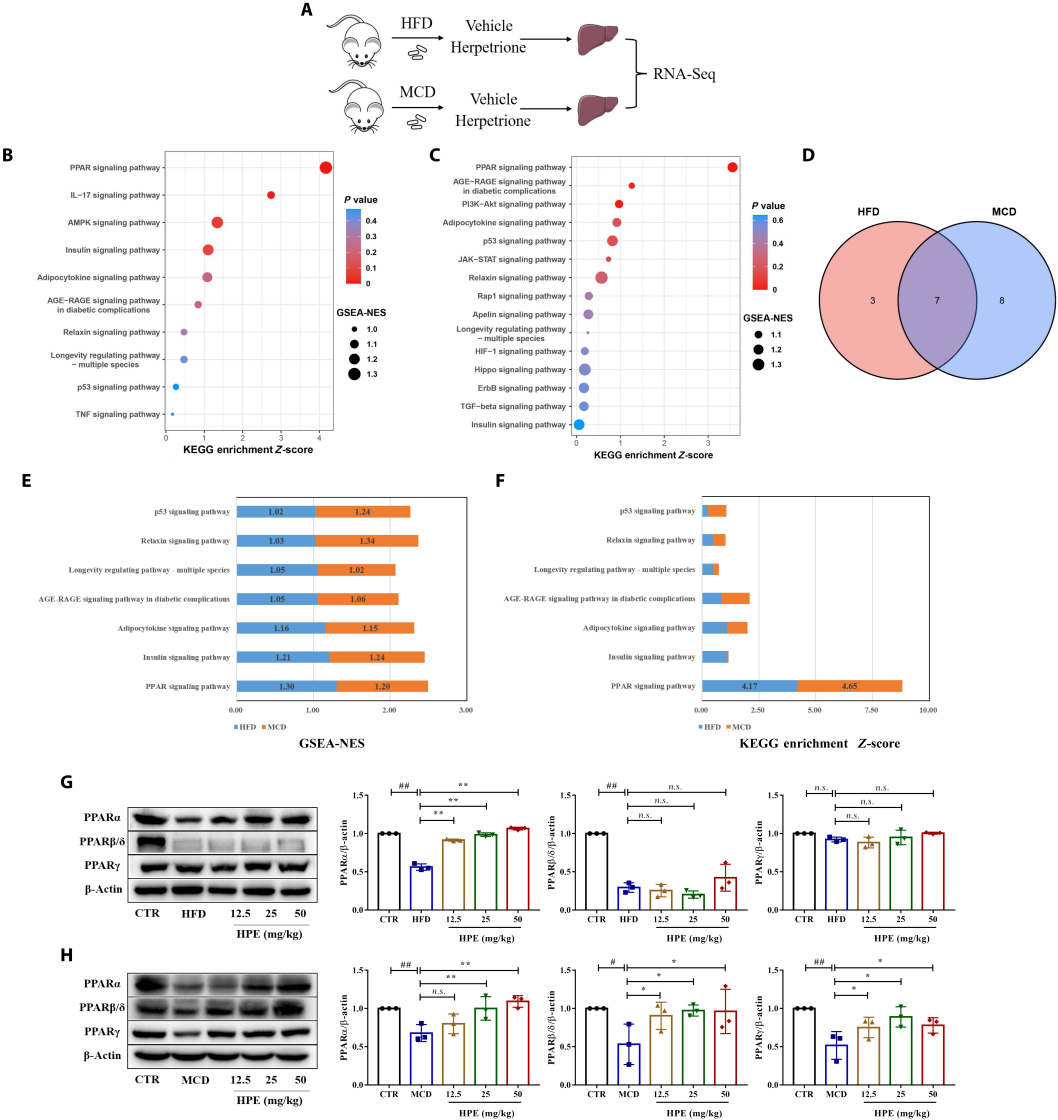
RNA-seq analysis shows that herpetrione boosts the activation of PPARα signaling in mice induced with HFD or MCD diet. (A) A procedure schematic for investigating the mechanism of herpetrione. (B and C) KEGG pathway enrichment analyses of transcriptomes from mouse liver samples induced with HFD (B) and MCD (C). (D to F) A Venn diagram (D) and corresponding scores, including GSEA-NES (E) and *Z*-score (F) of intersecting pathways based on transcriptomic data from HFD- and MCD-induced mice. (G and H) Protein expression of the PPAR signaling pathway was analyzed in mice that were fed either HFD (G) or were induced with MCD (H). Each group consisted of 3 mice and β-actin was used as a loading control.

RNA-seq analysis of the livers in mice was undertaken to investigate how herpetrione improves NASH caused by HFD and MCD feeding (Fig. 4A). Herpetrione was found to have regulatory effects on various physiological process, including lipid metabolism, glucose metabolism, inflammation, fibrosis, and apoptosis as per gene set enrichment analysis (GSEA) (Fig. S4), and heatmaps demonstrated that herpetrione partly restored the aberrant alterations in gene expression in the aforementioned pathway (Fig. S5). Further examination of the transcriptome data indicated 10 and 15 signaling pathways, respectively, in HFD-fed and MCD-fed mice (Fig. 4B and C). Among the 7 common signaling pathways, the PPAR pathway showed the highest score (Fig. 4D to F). A Western blot analysis was conducted on mice to examine the protein expression of PPARα, PPARβ, and PPARγ, which further confirmed herpetrione’s influence on the PPAR signaling pathway. Herpetrione significantly enhanced PPARα expression in both HFD- and MCD-fed mice (Fig. 4G and H), indicating that herpetrione could relieve NASH symptoms by activating PPAR signaling.

### Herpetrione can directly bind the PPARα protein

**Fig. 5.**
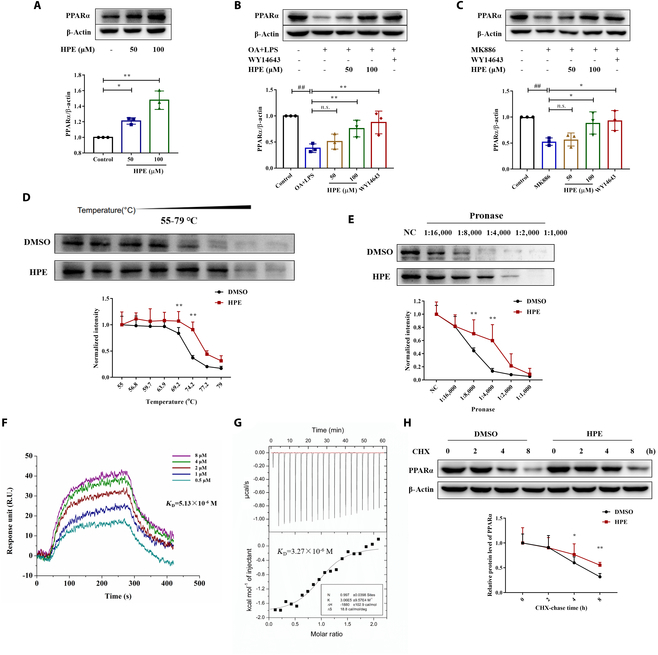
Herpetrione directly binds PPARα protein. (A) The expression level of PPARα protein was treated with DMSO and herpetrione at 50 μM or 100 μM in HepG2 cells. (B and C) Western blot analysis of PPARα protein in HepG2 cells stimulated by OA and LPS (B) and MK886 (C) in the presence or absence of herpetrione or WY14643. (D) CETSA analyzed the thermal stabilization of PPARα with herpetrione in HepG2 cell lysates. (E) DARTS analyzed the enzyme stabilization of PPARα with herpetrione in HepG2 cell lysates. (F) The interaction of PPARα with herpetrione was measured by SPR. (G) ITC enthalpogram of the interaction between herpetrione and PPARα. (H) Degradation of the PPARα protein was measured after the treatment of CHX at the indicated time points in HepG2 cells. β-Actin served as a loading control. Results are expressed as mean ± SD.

PPARα is responsible for regulating lipid catabolism and energy homeostasis. The Western blot analysis revealed that herpetrione promoted PPARα protein expression (Fig. 5A). Additionally, herpetrione activated PPARα protein expression in cells treated with OA combined with LPS (Fig. 5B) as well as in the presence of the PPARα-specific inhibitor MK886 (Fig. 5C). This activation is similar to WY14643, which acts as a potent PPARα agonist and is commonly referred to as pirinixic acid. Identifying small-molecule targets is critical for elucidating their mechanism of action. The cellular thermal shift assay (CETSA) analysis indicated that herpetrione shielded the PPARα protein against temperature-induced denaturation, thereby implying a direct interaction between herpetrione and PPARα (Fig. 5D). Drug affinity responsive target stability (DARTS) experiments substantiated the inhibitory effect of herpetrione on PPARα degradation caused by pronase (Fig. 5E). Additionally, surface plasmon resonance (SPR) analysis validated the interaction of herpetrione with PPARα, demonstrating an equilibrium dissociation constant (*K*_D_) of 5.13 μmol/L (Fig. 5F). In addition, the isothermal titration calorimetry (ITC) measurement also revealed an interaction between herpetrione and PPARα, with a *K*_D_ value of 3.27 μmol/L (Fig. 5G). These results corroborate that herpetrione is capable of directly binding to the PPARα protein. Cycloheximide (CHX), a protein synthesis inhibitor, has been used to determine the effect of herpetrione on PPARα protein stability. The results showed that the herpetrione weakened PPARα degradation compared to the control group (Fig. 5H); that is, herpetrione interacts with PPARα to improve the stability of the PPARα protein.

### Key amino acid residues in the interaction of herpetrione and PPARα

**Fig. 6.**
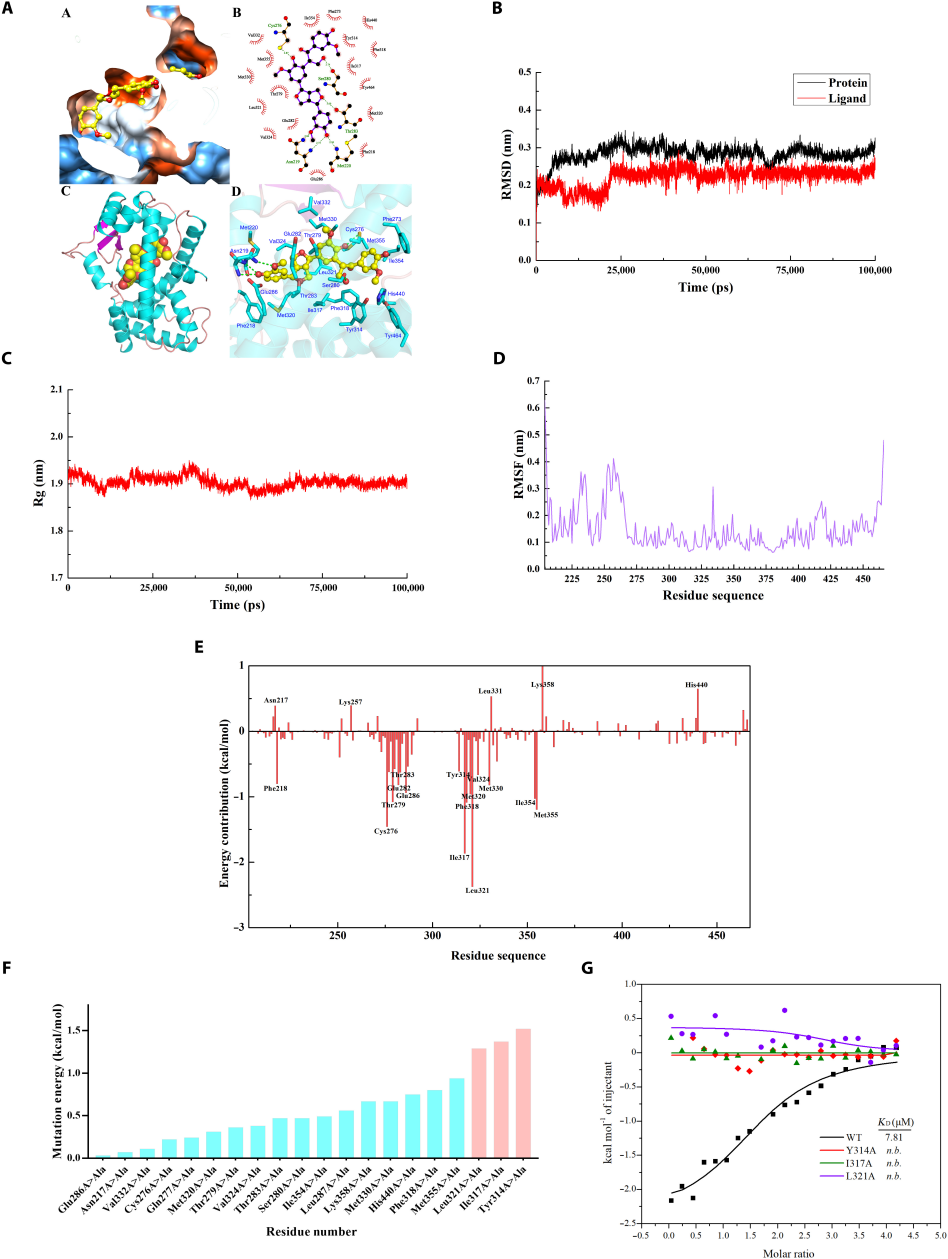
Key amino acid residues in the interaction of herpetrione and PPARα. (A) Molecular docking has predicted the binding of herpetrione with PPARα. (B to E) MD simulation showed the stable binding mode of herpetrione to PPARα. (B) Changes of RMSD values for backbone atoms of PPARα (black) and herpetrione (red). (C) Changes in Rg values. (D) RMSF values for protein Cα atoms were calculated. (E) Binding free energy decomposition of the PPARα–herpetrione complex. (F) ASM analysis of PPARα LBD in complex with herpetrione. (G) ITC enthalpogram of the interaction between herpetrione and PPARα-WT, Y314A, I317A, or L321A, respectively.

We next investigated the interaction mode between herpetrione and PPARα, focusing on the crucial amino acid residues. Molecular docking analysis showed the presence of Phe273, Cys276, Gln277, Thr279, Ser280, Thr283, Tyr314, Ile317, Leu321, Met330, Ile354, Met355, His440, Val444, and Tyr464 in the binding pocket (Fig. 6A). Subsequently, molecular dynamics simulation was used to investigate the stability of the interaction between herpetrione and PPARα. Root mean square deviation (RMSD) values of PPARα and herpetrione fluctuated by approximately 0.3 and 0.25 nm, respectively, and the radius of gyration (Rg) values were all approximately 1.9 nm, indicating that the complex remained stable and compact (Fig. 6B and C). Furthermore, the equilibrium trajectory from 87 ns to 92 ns was chosen to simulate the protein–ligand interactions. The root mean square fluctuation (RMSF) values of most amino acid residues in the binding pocket were within 0.2 nm, suggesting the steady condition of the binding pocket in molecular dynamics simulations (Fig. 6D).

In addition, the binding free energy decomposition calculation in the PPARα–herpetrione complex was performed using the MM/PBSA method to investigate the critical residues responsible for herpetrione binding. The results showed that some residues played an important role in ligand binding, including Leu321, Ile317, Cys276, Met355, Phe318, Thr279, and Ile354, and the absolute value of their contribution to binding free energy exceeded 1.0 kcal/mol (Fig. 6E). Consistently, the data of alanine scanning mutagenesis (ASM) indicated marked changes in the binding free energy due to certain mutations. Among them, Tyr314, Ile317, and Leu321 were the 3 amino acid residues with the greatest variation in mutation energy, with values of 1.52, 1.37, and 1.29 kcal/mol, respectively (Fig. 6F). Based on the virtual analysis, Tyr314, Ile317, and Leu321 were mutated to alanine to purify the constructed PPARα protein. Further ITC results showed that the *K*_D_ of the PPARα WT protein was 7.81 μmol/L, while mutations at Tyr314, Ile317, and Leu321 resulted in herpetrione losing the ability to bind PPARα (Fig. 6G and Fig. S6). In summary, Tyr314, Ile317, and Leu321 were the key sites of herpetrione for binding with PPARα.

### The protective effects of herpetrione against lipid accumulation and inflammation in hepatocytes are attributable to the activation of PPARα

**Fig. 7.**
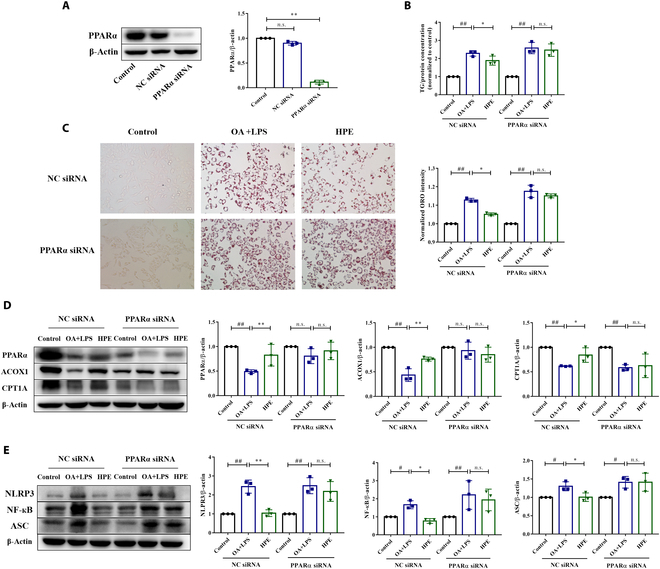
The protective effects of herpetrione on lipid accumulation and inflammation in hepatocytes are attributed to PPARα activation. (A) Test of knockdown efficacy of PPARα by RNA interference in HepG2 cells. (B) Cellular TG content of HepG2 cells. (C) Representative ORO staining images in HepG2 cells treated with DMSO or herpetrione before and after stimulation with OA and LPS, in the presence or absence of PPARα siRNA, were observed. The magnification is set to 200×. (D and E) Protein level of lipid metabolism (D)- and inflammation (E)-associated molecules in HepG2 cells. *n* = 3 per group. β-Actin served as a loading control. The data are presented as the mean ± SD.

To investigate whether herpetrione mediates hepatoprotective effects in a manner dependent on the regulation of PPARα activation, PPARα was knocked down in HepG2 cells using siRNA (Fig. 7A). Herpetrione treatment significantly reduced intracellular TG levels, but this effect was mitigated by PPARα siRNA (Fig. 7B). In addition, ORO staining revealed that the ability of herpetrione to lower lipid droplet accumulation was almost abolished owing to PPARα knockdown (Fig. 7C). To examine whether PPARα plays a crucial role in the lipid-lowering and anti-inflammatory effects of herpetrione, we conducted Western blot. This assessed the expression of metabolic lipids and inflammatory molecules in the presence and absence of siRNA. PPARα siRNA resulted in a decline in the herpetrione-induced enhancement of fatty acid oxidation (Fig. 7D). Similarly, reductions in inflammatory molecule expression were abrogated (Fig. 7E). These findings suggest that the shielding influence of herpetrione on lipid buildup and inflammation during OA and LPS provocation was nullified by PPARα insufficiency.

### The regulatory effect of herpetrione on liver steatosis and inflammation is PPARα dependent in mice

**Fig. 8.**
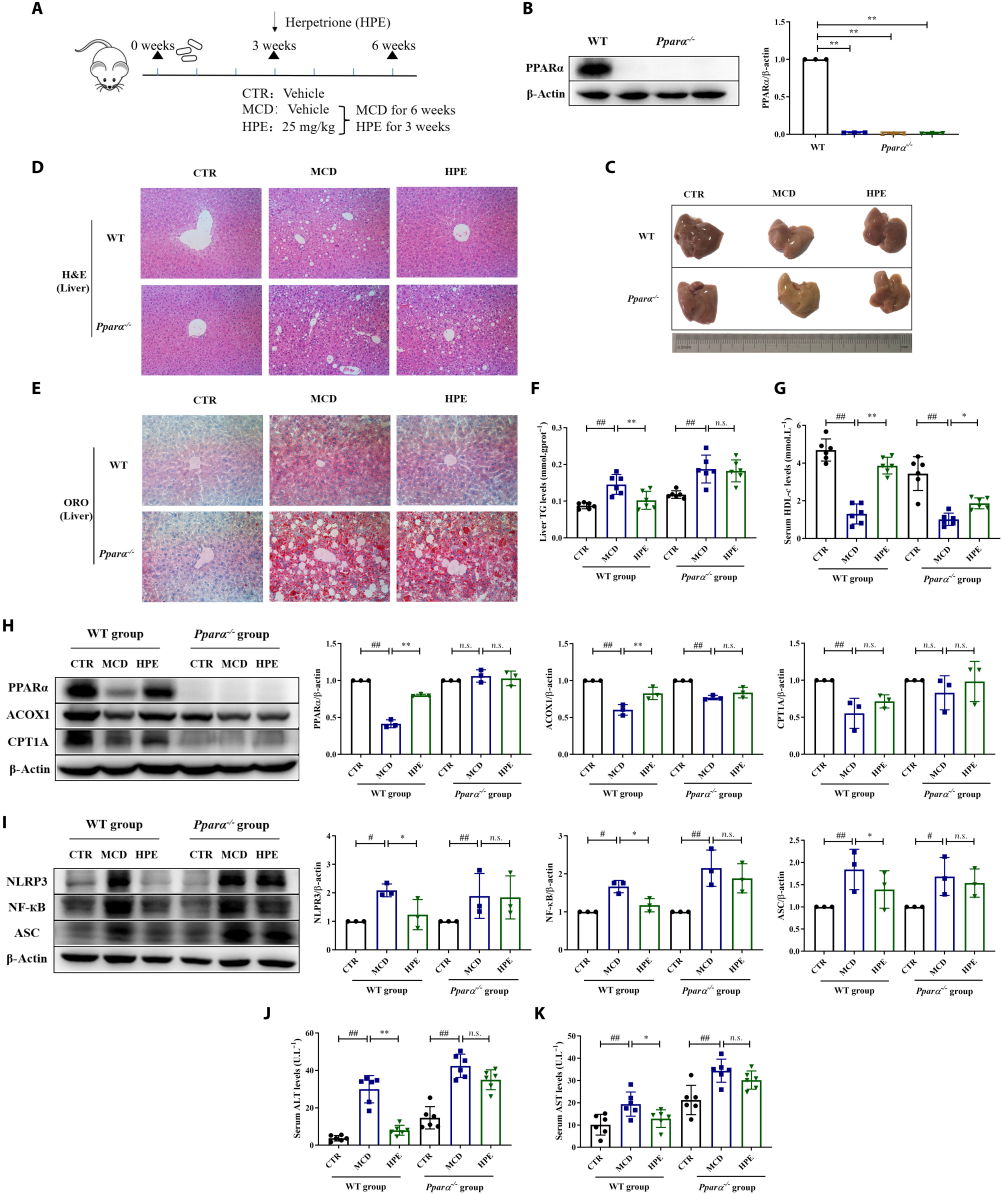
The regulatory role of herpetrione on lipid accumulation and inflammation is dependent on PPARα in mice. (A) Schematic of the experimental process for assessing the impact of herpetrione on MCD-induced NASH in wild type and PPARα knockout mice, with 6 individuals in every group. (B) Hepatic PPARα protein level in wild-type mice and PPARα knockout mice. (C) Representative images of liver morphology. (D and E) H&E staining (D) and ORO staining images (E) of liver sections from MCD-fed indicated mice (200× magnification). (F and G) TG contents (F) and HDL-c contents (G) in mice. (H and I) Protein expression levels of molecules associated with lipid metabolism (H) and inflammation (I) were assessed in the livers of the indicated mice. (J and K) Serum concentrations of ALT (J) and AST (K) were measured in the mice as indicated. β-Actin served as a loading control. Results are expressed as mean ± SD.

To assess whether PPARα activation is necessary for the anti-NASH effects of herpetrione, mice were fed an MCD diet for 3 weeks. Subsequently, for a further 3 weeks, they received daily doses of herpetrione while still consuming the MCD diet (Fig. 8A). Both wild-type and PPARα knockout mice were employed in this study. The Western blot analysis illustrated that the PPARα protein was detectable in the livers of wild-type mice but absent in those of knockout mice (Fig. 8B). H&E and ORO staining demonstrated that the relief of hepatic lipid accumulation induced by herpetrione ceased due to PPARα deficiency (Fig. 8C to E). The reductions in TG levels were also annulled in mice with PPARα knockout (Fig. 8F). Moreover, a significant attenuation of the considerable rise in HDL-c levels was noted in PPARα knockout mice, as opposed to wild-type mice (Fig. 8G). Consistently, the regulatory effects of herpetrione on fatty acid oxidation and the inflammatory response were eradicated in PPARα knockout mice (Fig. 8H and I). The reduction in ALT and AST levels, the distinguishing enzymes of liver injury caused by herpetrione, was also eliminated due to the absence of PPARα (Fig. 8J and K). These findings collectively indicate that the effectiveness of herpetrione in NASH mice is entirely abolished by PPARα deficiency; this suggests that the regulatory impact of herpetrione on NASH is dependent on the PPARα signal.

## Discussion

The burden associated with NASH and its mortality rate are rapidly increasing; thus, this disease poses a major public health problem. PPARα is involved in glucolipid metabolism as well as inflammation and fibrosis; therefore, it is generally considered a therapeutic target for NASH [11,25]. In this study, herpetrione effectively halted NASH progression in both hepatocytes and mice. Herpetrione also promoted PPARα signaling activation while directly binding to the PPARα protein. Furthermore, the protective effects of herpetrione against NASH were largely eliminated by PPARα deficiency, implying that herpetrione could be utilized as a therapeutic agent for NASH.

Steatosis is typically caused by excessive calorie consumption and an accumulation of TGs, which is a major characteristic of NASH [26]. The majority of the free fatty acids that are esterified to form hepatic TGs mainly originate from adipose tissue lipolysis, but can also result from dietary fat intake and de novo lipogenesis [27]. The bulk of ingested fat is usually directed toward adipose tissue or metabolic use in muscles, either for storage or oxidation. However, lipolysis in white adipose tissue causes the release of fatty acids, which can lead to an excessive storage of TG in hepatocytes, resulting in liver steatosis [28,29]. In this study, it was found that herpetrione can reduce hepatic lipid levels in hepatocytes stimulated with OA and LPS and in the livers of mice fed with HFD or MCD diet. Additionally, herpetrione was observed to inhibit the expression of SREBP1 and FASN proteins while promoting the expression of PPARα, ACOX1, and CPT1A proteins in vitro and in vivo. This suggests that herpetrione may improve hepatic steatosis by inhibiting the de novo synthesis and uptake of fatty acids while enhancing fatty acid β-oxidation. Indeed, it has been shown that NASH patients exhibit hepatic de novo lipogenesis to compensate for the increase in fatty acid influx to the liver [30]. In addition, it is known that NASH is causally linked to insulin resistance, generally through obesity [31]. Administration of herpetrione alleviated fasting blood glucose and insulin levels induced by HFD via the IRS1/AKT/FOXO1 signaling pathway, and lowered the HOMA-IR index.

Another feature of NASH is the manifestation of liver inflammation, which is believed to contribute to the formation of fibrosis [32]. Previous studies have shown that *H. caudigerum* contains lignan components that exhibit free radical-scavenging, antioxidant, and anti-inflammatory effects [33,34]. Moreover, research has shown that *H. caudigerum* is capable of reducing the MDA content in the liver, increasing the superoxide dismutase content, and inhibiting the expression of NF-κB. This suggests that *H. caudigerum* may have a positive effect on liver function by enhancing the anti-inflammatory capacity [35]. Furthermore, in our study, herpetrione ameliorated NASH via inhibition of NF-κB nucleation and NLRP3 activation, and suppression of inflammatory cytokine secretion induced by metabolic stress. In addition, herpetrione showed a reduction in NASH-associated fibrosis as observed through Sirius red staining analysis.

To clarify the potential mechanism by which herpetrione alleviates NASH, an RNA-seq analysis was conducted, which revealed that the PPAR signaling pathway had higher scores than other pathways. Additionally, herpetrione considerably stimulated the activation of the PPARα signaling pathway, which is closely involved in the metabolism of lipids, glucose, amino acids, and inflammation, and is a critical sensor and regulator of lipids. Upon the activation of PPARα, TG clearance and HDL cholesterol levels are substantially increased; these changes were also observed in our study. PPARα is a ligand-inducible transcription factor, and some high-affinity PPARα agonists are currently being tested as potential treatments for NASH [36,37]. In this study, the binding affinity between herpetrione and PPARα was detected, with cracking *K*_D_ value. Natural products have long been a promising source for drug discovery, and some natural products that have traditionally been used for liver protection are rich in lignans. Among these, honokiol and magnolol, both neolignans derived from *Magnolia officinalis*, improve glucose and lipid metabolism in HFD-induced mice by activating PPARγ [38]. Furthermore, honokiol was identified as a partial PPARγ activator and found to directly bind to PPARγ [39]. However, there has been no research conducted on the capacity of lignans to target PPARα. This study is, to the best of our knowledge, the first to establish that herpetrione, a lignan, directly binds to PPARα.

Further data confirmed that Tyr314, Ile317, and Leu321 were the key binding sites of herpetrione to PPARα. Previous studies have shown that the PPARα-LBD pocket is capacious enough to accommodate multiple ligands with diverse structures, such as naturally occurring fatty acids, synthetic drugs like fibrates, and the investigational drug WY14643 [40]. The Arm III domain is crucial for enhancing PPARα activity and selectivity. PPARα agonists, which bind more deeply in Arm III, may have stronger effects [41]. The critical binding sites of herpetrione when binding PPARα, Leu321, and Ile317, extend into the Arm III domain. Similarly, the binding sites between PPARα and pemafibrate, also known as K-877 with higher efficacy in reducing TG levels compared to other fibrates, expand into the Arm III domain [42]; this suggests that herpetrione could be a potential PPARα ligand for managing NASH. Our research provides insight into the effectiveness of a natural product in treating NASH as well as the broader prospects for NASH treatment by targeting PPARα.

## Materials and Methods

### Chemicals and reagents

Herpetrione was isolated and identified from *H. caudigerum* on the basis of the ^1^H and ^13^C nuclear magnetic resonance spectra (Figs. S7 and S8) with support of a check of purity by high-performance liquid chromatography. The primary antibodies were purchased from Proteintech (Wuhan, China) and Beyotime (Shanghai, China); this cat. no. was provided in the Supplementary Materials (Table S1).

### Cell culture and treatment

HepG2 and AML12 cells were obtained from the Cell Bank of Type Culture Collection of the Chinese Academy of Sciences (Shanghai, China). Cells were cultured in Dulbecco’s Modified Eagle Medium (DMEM), which was supplemented with 10% fetal bovine serum (10099-141; Gibco, USA) and 1% penicillin-streptomycin (15140-122; Gibco, USA) and placed in a 5% CO_2_ incubator at 37 °C. The cells underwent serum starvation in DMEM without serum for 12 h prior to an additional 24-h incubation with 0.2 mM OA (O-1008; Sigma-Aldrich, USA) and 2 μg/ml LPS (S1732; Beyotime, China) in the absence or presence of herpetrione (50 to 200 μM).

### Methylthiazolyltetrazolium analysis

Cells were seeded in 96-well plates. After adhesion, the cells were incubated with different concentrations of herpetrione for 24 h. Afterward, methylthiazolyl tetrazolium was added to each well, and cells were sequentially incubated for 4 h at 37 °C. To dissolve the formazan, dimethyl sulfoxide (DMSO) was added, and the microplate reader (Synergy h1, BioTek, USA) measured the absorbance at 490 nm.

### Cellular oil red O staining

Cells were fixed in 4% paraformaldehyde for 20 min. After that, the cells were immersed in 60% isopropanol solution, and subsequently stained with Oil Red O dye (G1262; Solarbio, China) for 30 min. Images were captured using a light microscope (Ti-S, Nikon, Japan).

### Transfection of siRNA

For gene knockdown, chemically synthesized siRNA targeting PPARα was procured from Tsingke, China. HepG2 cells were transfected with siRNA using Lipofectamine 2000 in accordance with the manufacturer’s instructions from Invitrogen, USA. The efficiency of knockdown was determined via Western blot analysis.

### Animal experiments

All animal welfare and experimental procedures received approval from the Institutional Animal Care and Use Committee of Southwest University in China. We purchased male C57BL/6 mice from Ensiweier Co., Ltd., while *Pparα*^−/−^ mice (on the C57BL/6 background) were bought from Shanghai Model Organisms Center, Inc. The mice were all 8 weeks old and were housed under a 12-h light–dark cycle with unrestricted access to food and water. The temperature was maintained at 24 ± 2 °C with a relative humidity of 45%. NAFLD and NASH models were formed by continuously feeding mice an HFD (D12492; protein, 20%; fat, 60%; carbohydrates, 20%; Beijing, China) or MCD (AIN-76; protein, 17%; fat, 10%; carbohydrate, 66%; Jiangsu, China) for 24 weeks or 8 weeks, respectively. Mice in the control group were fed a normal chow (NC) diet (D12451, Beijing, China; MCS, Jiangsu, China). Mice received herpetrione (12.5 to 50 mg/kg) via intragastric gavage daily, with vehicle (blank solution) used as a control. To investigate the involvement of PPARα in herpetrione-mediated improvement in NASH, *Pparα*^−/−^ mice were subjected to an MCD diet for 6 weeks, as previously described.

### Histological analysis

Tissues were fixed overnight using a 10% formaldehyde solution. Standard protocols were followed for the H&E and ORO staining of paraffin-embedded and compound-embedded frozen liver sections. Histological images of tissue sections were captured using a light microscope (Ti-S, Nikon, Japan). For immunohistochemistry, paraffin sections of mouse liver were deparaffinized, rehydrated, blocked, and immunostained with anti-F4/80 antibodies (Servicebio, China) at 4 °C overnight. Afterward, the slides were incubated with a secondary antibody for 60 min, followed by visualization of the sections using light microscopy (Ti-S, Nikon, Japan).

### Serum enzyme and lipid assays

The levels of ALT, AST, LDL-c, HDL-c, glucose tolerance, and insulin tolerance test present in serum were determined utilizing microplate reader (A Synergy h1; BioTek, USA), following the instructions provided. Measurement of TG and TC lipid levels in cells or the liver was performed through commercially available kits. Subsequently, inflammatory cytokine levels in the serum were measured using enzyme-linked immunosorbent assay kits.

### Western blot

Tissues or cells were homogenized in radio immunoprecipitation assay lysis buffer containing protease and phosphatase inhibitors, which was kept ice-cold. Lysates were separated by 8% to 12% sodium dodecyl sulfate-polyacrylamide gel electrophoresis (SDS-PAGE) and transferred to polyvinylidene difluoride membranes (IPVH00010; Millipore, USA). The membranes were then blocked in 5% nonfat milk and probed with appropriate primary antibodies overnight at 4 °C. The membranes were further incubated with secondary antibodies for 2 h at room temperature. Finally, membranes were treated with an ECL kit (PK10002; Proteintech, China), and images were recorded (Tanon5200; Shanghai, China).

### Cellular thermal shift assay

The cell lysates were divided into 2 aliquots; one aliquot acted as a control, whereas the other was incubated with herpetrione for 1 h at room temperature. Next, the lysates were heated individually at the temperatures stated (55 to 79 °C) for 5 min and allowed to cool at ambient temperature. The lysates were then centrifuged at 12,000 rpm for 10 min at 4 °C, after which, the supernatants were analyzed by Western blot.

### Drug affinity responsive target stability

At room temperature, the cell lysates were mixed with herpetrione for 30 min. Pronase (1:1,000 to 1:16,000) was subsequently added, and the samples were incubated at 40 °C for 30 min. The reactions were stopped by adding loading buffer and analyzed via immunoblotting following SDS-PAGE separation.

### Protein expression and purification

The human PPARα ligand binding domain was expressed as an N-terminal 6×His fusion protein induced by the vector pET24a. The production of recombinant proteins was achieved by transforming the expression plasmids into *Escherichia coli* BL21 (DE3) cells. Cells were cultivated to an OD_600_ of 0.6 to 0.8 and treated with 0.5 mM isopropyl-β-D-thiogalactoside at 18 °C for 13 h. The cells were subsequently isolated through centrifugation at 7,000 rpm for 10 min at 4 °C and lysed in 40 ml of lysis buffer (0.01 M PO_4_^3−^, 0.8% NaCl, 0.02% KCl, 10% Tween 20, 0.3 μg/ml lysozyme, and 0.1% 2-hydroxy-1-ethanethiol). Following that, proteins were purified utilizing Ni-nitrilotriacetic acid resin (GE Healthcare, USA) with phosphate buffered solution (PBS) buffer (a gradient of 10 to 400 mM imidazole). The proteins were concentrated using Amicon Ultra 10K centrifugal filtration (Millipore, USA) and subsequently dissolved in PBS buffer. Verification of the presence of purified proteins occurred through Coomassie brilliant blue staining prior to use.

### Isothermal titration calorimetry

ITC was conducted at 25 °C using a MicroCal ITC200 microcalorimeter. The proteins were dialyzed extensively in PBS buffer, and the dialysis buffer was utilized for the dilution of ligand stock solutions. In addition, DMSO was added to the protein solution at the identical concentration present in the ligand solution. The sample cell was preloaded with PPARα protein (25 μM), and herpetrione (500 μM) was pre-injected into the pipette. A reference titration of the ligand into buffer was used to correct for the heat of dilution. The syringe stirring speed was set to 1,000 rpm. The thermodynamic data were analyzed using MicroCal’s Origin 7.0 software.

### Surface plasmon resonance

The interaction between herpetrione and PPARα was examined with an OpenSPR instrument (Nicaya, Canada). The carboxyl sensor chip (SEN-AU-100-3-COOH; Nicaya, Canada) was utilized to immobilize the recombinant PPARα protein via a standard amine coupling method. Analytes consisting of various concentrations of herpetrione (0.5 to 8 μM) within the running buffer were injected at a 20 μl/min flow rate. The interaction parameter *K*_D_ was assessed using TraceDrawer evaluation software according to the 1:1 Langmuir model.

### CHX chase assay

Cells were incubated with herpetrione for 24 h. Subsequently, they were treated with 50 μg/ml CHX (S7418; Selleck, USA) and samples were collected at 0, 2, 4, and 8 h. After that, the samples were lysed to harvest proteins for subsequent Western blot analysis.

### RNA sequencing and bioinformatic analysis

Total RNA was extracted, and cDNA libraries were created to examine disparities in gene expression. The Illumina sequencing platform was utilized for single-end sequencing of libraries. HISAT2 software (version 2.0.5) was utilized to map the reads to the mouse reference genome in Ensembl. StringTie (version 1.3.3b) was used to calculate raw counts of genes. DESeq2 (version 1.20.0) software was applied to normalize the count matrix.

### Gene set enrichment analysis

GSEA was undertaken using the Java GSEA platform. For each biological pathway in the GO and KEGG analysis, the genes comprising gene set were identified and a ranked list was generated, accompanied by a “gene set” permutation. Gene sets with a *P* value of less than 0.05 were deemed to have significant differential expression.

### Molecular docking analysis

The crystal structure of PPARα (Protein Data Bank ID: 4BCR) was obtained from a protein database, and the protein was subjected to energy minimization as the receptor structure for molecular docking. The substrate of herpetrione was constructed and hydrogenated, and the molecular orbital package program was used to optimize the structure. Molecular docking was carried out using Autodock software; the docking box was wrapped around the active site, and the docking time was set to 100. The default value was used for all other parameters.

### Molecular dynamics simulation and analysis

Gromacs was used to do a 100-ns molecular dynamics simulation to study the interaction mode between herpetrione and PPARα. The protein–ligand complex reference structures were derived from the binding structures predicted by molecular docking. The complex’s RMSD, Rg, and RMSF values were determined using the commands gmx rms, gmx gyrate, and gmx rmsf, respectively. To explore the essential residues for herpetrione and PPARα binding, the binding free energy breakdown of the PPARα–herpetrione complex was computed using the MM/PBSA method.

### Virtual amino acid mutation analysis

The lowest-energy conformation in the molecular dynamics simulation was selected to calculate the mutation energy of the complex by the ASM assay in Discovery Studio 2021 software to evaluate the importance of a certain amino acid residue for the binding of herpetrione and PPARα.

### Statistical analysis

All data are presented as mean ± SD. Statistical analysis and graphing were undertaken using GraphPad Prism 9.0. Mean values were compared using one-way analysis of variance (ANOVA) with Dunnett’s post hoc test, and significant differences between 2 groups were analyzed using the Student’s *t* test. *P* values are expressed as follows: *P < 0.05; **P < 0.01; #*P* < 0.05, ##*P* < 0.01; n.s., not significant.

## Data Availability

All data are available in the main text or the Supplementary Materials. Additional data related to this paper may be requested from the authors.
