# Supplementary material for: Herpetrione, a New Type of PPARα Ligand as a Therapeutic Strategy Against Nonalcoholic Steatohepatitis
Source: Research (Wash D C). 2023 Nov 30;6:0276. doi: 10.34133/research.0276 (PMC10687582; doi:10.34133/research.0276)
Supplement: Supplementary 1 — Figs. S1 to S8 Table S1 [file research.0276.f1.zip › Supplemental Material.docx]

Supplementary Materials

**Tab. S1** Antibody information

**Fig. S1** Herpetrione lessens lipid accumulation and inflammation in AML12 cells encouraged by OA and LPS

**Fig. S2** Herpetrione inhibits HFD-induced obesity and inflammation

**Fig. S3** Herpetrione inhibits hepatic steatosis and inflammation in MCD mice

**Fig. S4** Herpetrione regulates glucose metabolism, lipid metabolism and inflammation in HFD- or MCD- induced mice based on transcriptomic analysis

**Fig. S5** Herpetrione adjusts gene expression profiles using the RNA-seq dataset in mice that were induced with either HFD or MCD

**Fig. S6** Herpetrione cannot effectively bind to the PPARα mutant protein

**Fig. S7** ^1^H NMR (400 MHz, methanol-*d_4_*) spectrum of herpetrione

**Fig. S8** ^13^C NMR (400 MHz, methanol-*d_4_*) spectrum of herpetrione

**Tab. S1:** Antibody information

| **Antibody Target** | | **Cat. No.** | **Manufacturer** |
| --- | --- | --- | --- |
| **PPARα** | 66826-1-Ig | Proteintech |  |
| **PPARβ/δ** | 10156-2-AP | Proteintech |  |
| **PPARγ** | 16643-1-AP | Proteintech |  |
| **CPT1A** | 15184-1-AP | Proteintech |  |
| **FASN** | 10624-2-AP | Proteintech |  |
| **ACOX1** | AF6129 | Beyotime |  |
| **SREBP1** | AF8055 | Beyotime |  |
| **NLRP3** | 19771-1-AP | Proteintech |  |
| **ASC** | 10500-1-AP | Proteintech |  |
| **Caspase 1** | 22915-1-AP | Proteintech |  |
| **NF-**κ**B** | 10745-1-AP | Proteintech |  |
| **IKKα** | AF0198 | Beyotime |  |
| **IκB** | AF1282 | Beyotime |  |
| **IRS1** | AF7299 | Beyotime |  |
| **p-IRS1** | AI623 | Beyotime |  |
| **FOXO1** | AF1600 | Beyotime |  |
| **p-FOXO1** | AF5824 | Beyotime |  |
| **AKT** | 60203-2-Ig | Proteintech |  |
| **p-AKT** | 80445-1RR | Proteintech |  |
| **Keap1** | 10503-2-AP | Proteintech |  |
| **Nrf2** | 16396-1-AP | Proteintech |  |
| **HO1** | 10701-1-AP | Proteintech |  |
| **NQO1** | 11451-1-AP | Proteintech |  |
| **β-actin** | 81115-1-RR | Proteintech |  |

**Supplementary figure legends**

**Fig. S1** Herpetrione reduces lipid accumulation and inflammation in AML12 cells stimulated with OA and LPS. (A) Cytotoxicity of herpetrione in AML12 cells. (B) Detection of TG contents in AML12 cells treated with DMSO, 100 μM, or 200 μM herpetrione was conducted in response to OA and LPS over a 24-hour period. (C) ORO staining of lipid droplets in AML12 cells. The magnification is set to 200×. (D) Representative western blot of lipid metabolism-related molecules (PPARα, ACOX1, CPT1A, SREBP1 and FASN) in AML12 cells. (E) Representative western blot of inflammation-related molecules (NF-κB, IKKα and IκB) in AML12 cells. n = 3 per group. β-actin served as a loading control. The data are presented as the mean ± SD.

**Fig. S2** Herpetrione restrains HFD-induced obesity and inflammation. (A-B) White adipose weight (A) and the ratio of white adipose weight and body weight (B) in the indicated groups. n = 8 per group. (C) Body weight of mice in the indicated groups. (D-E) Hepatic TC (D) and serum LDL-_C_ (E) contents of the mice in the indicated groups. (F) H&E staining of white adipose tissue and F4/80 staining of liver in mice. The magnification is set to 200×. Results are expressed as mean ± SD.

**Fig. S3** Herpetrione suppresses hepatic steatosis and inflammation in mice fed an MCD diet. (A-B) Liver weight (A) and the ratio of liver weight and body weight (B) in the indicated groups. n = 8 per group. (C) Body weight of mice in the indicated groups. (D-E) Hepatic TC (D) and serum HDL-_C_ (E) contents of the mice in the indicated groups. (F) F4/80 staining and sirius red staining of liver in mice. The magnification is set to 200×. Results are expressed as mean ± SD.

**Fig. S4** Herpetrione regulates glucose metabolism, lipid metabolism and inflammation in HFD- or MCD- induced mice based on transcriptomic analysis. (A) GSEA analysis was conducted on pathways concerning lipid and glucose metabolism, as well as inflammation and apoptosis, in mice induced with HFD. (B) In mice induced with MCD, GSEA analysis was performed on pathways related to lipid metabolism, inflammation, fibrosis, and apoptosis.

**Fig. S5** Herpetrione adjusts gene expression profiles according to the RNA-seq data set in HFD- or MCD- induced mice. (A) Illustration of pathways related to lipid metabolism, glucose metabolism, inflammation and apoptosis in HFD-induced mice in the form of a heatmap. (B) Illustration of pathways related to lipid metabolism, inflammation, fibrosis, and apoptosis in MCD-induced mice in the form of a heatmap.

**Fig. S6** Herpetrione cannot effectively bind to the PPARα mutant protein. ITC enthalpogram of the interaction between herpetrione and PPARα-WT, Y314A, I317A, or L321A mutant protein, respectively.

**Fig. S7** ^1^H NMR (400 MHz, methanol-*d*_4_) spectrum of herpetrione.

**Fig. S8** ^13^C NMR (400 MHz, methanol-*d*_4_) spectrum of herpetrione.
