# Supplementary figures and images for: Herpetrione, a New Type of PPARα Ligand as a Therapeutic Strategy Against Nonalcoholic Steatohepatitis
Source: Research (Wash D C). 2023 Nov 30;6:0276. doi: 10.34133/research.0276 (PMC10687582; doi:10.34133/research.0276)

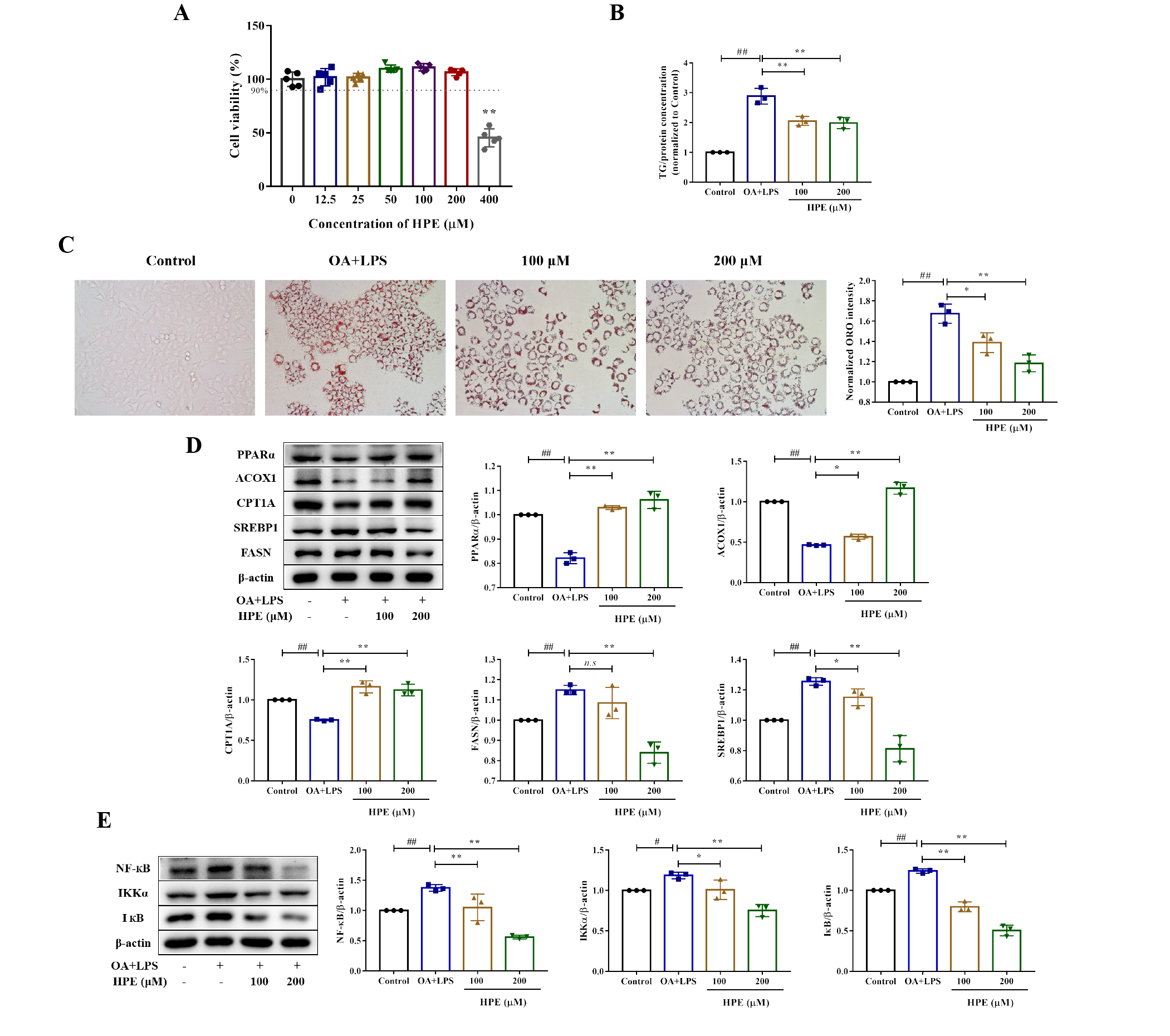

Supplement: Supplementary 1 — Figs. S1 to S8 Table S1 [file research.0276.f1.zip › Figure-S1.tif]

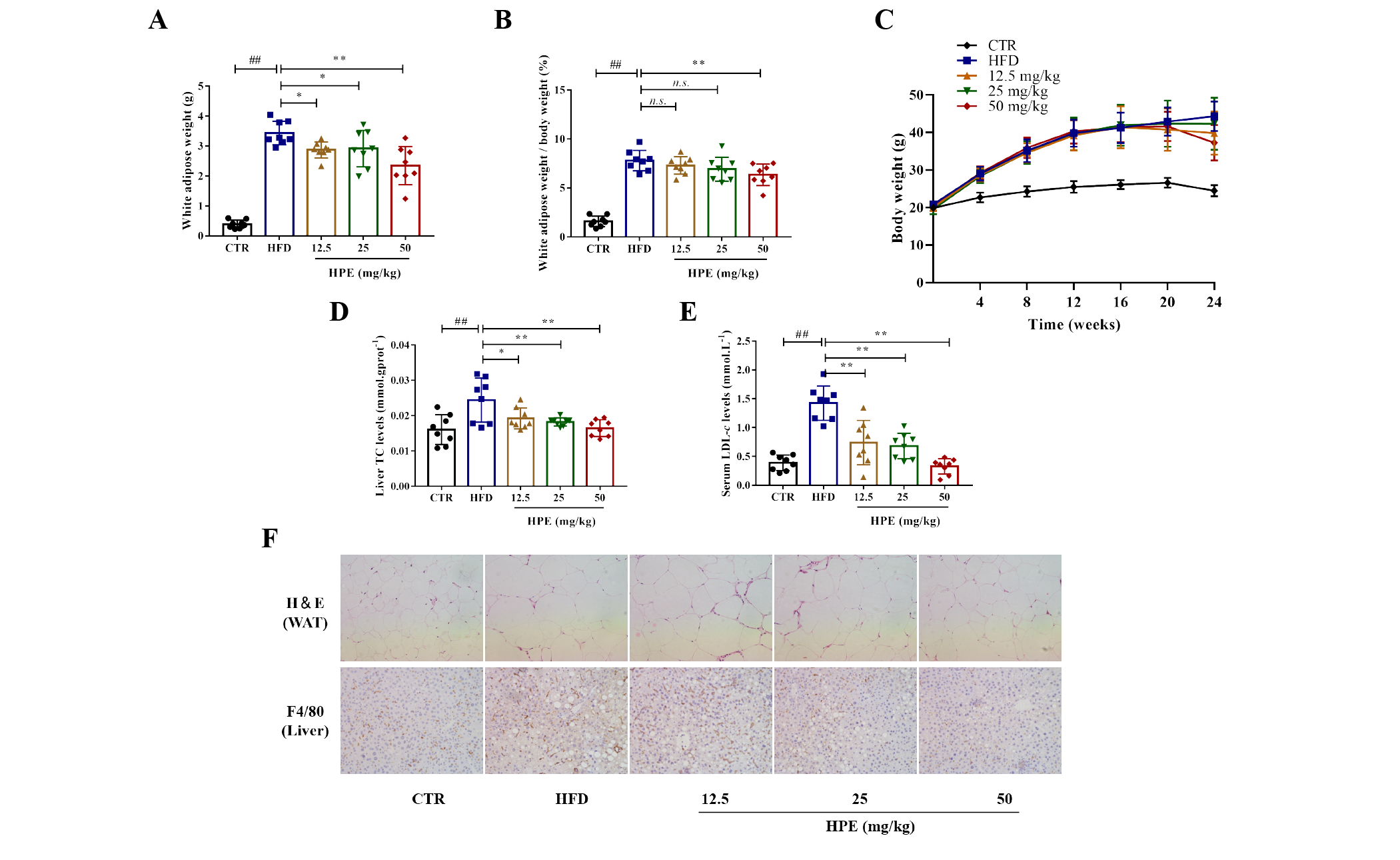

Supplement: Supplementary 1 — Figs. S1 to S8 Table S1 [file research.0276.f1.zip › Figure-S2.tif]

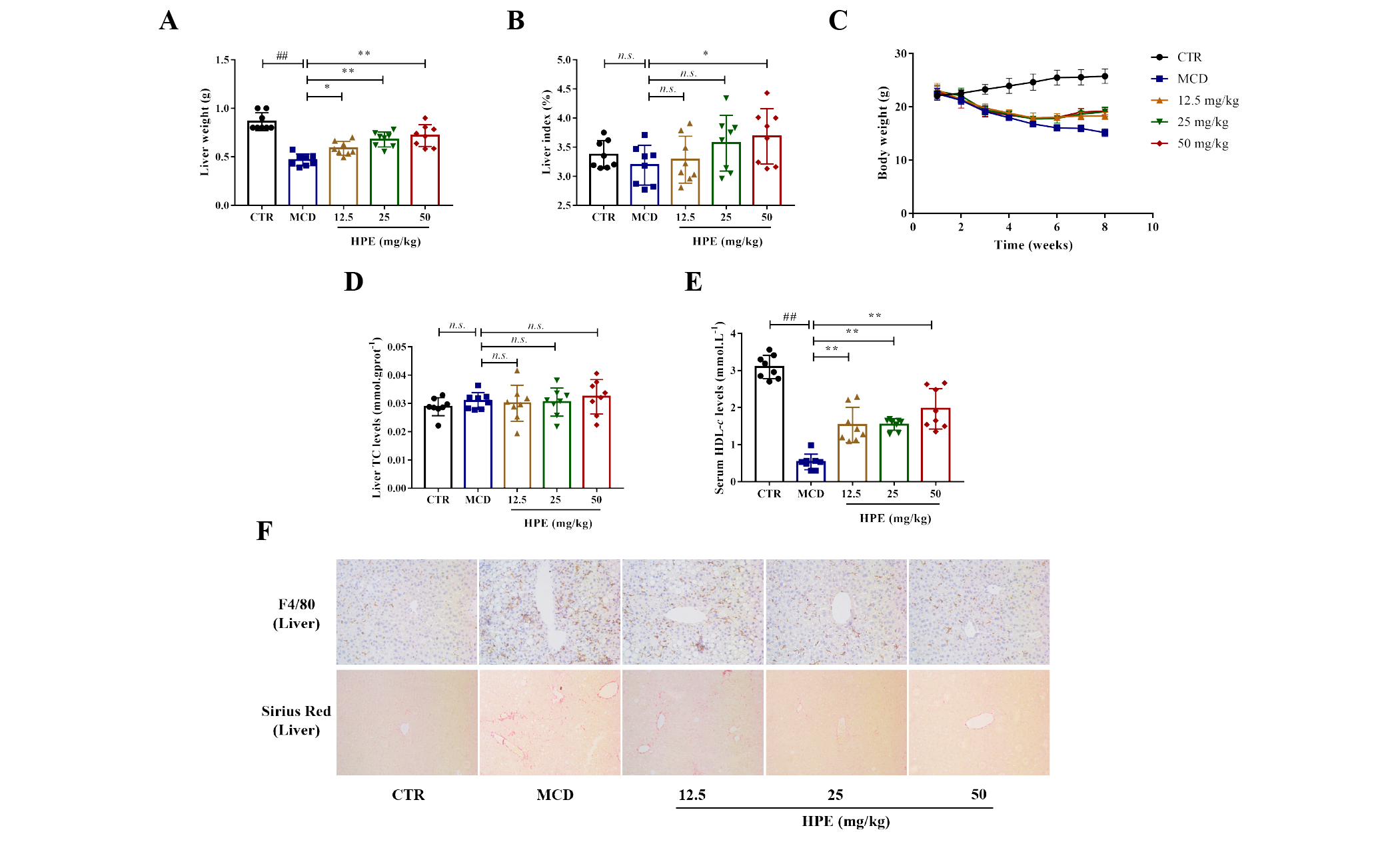

Supplement: Supplementary 1 — Figs. S1 to S8 Table S1 [file research.0276.f1.zip › Figure-S3.tif]

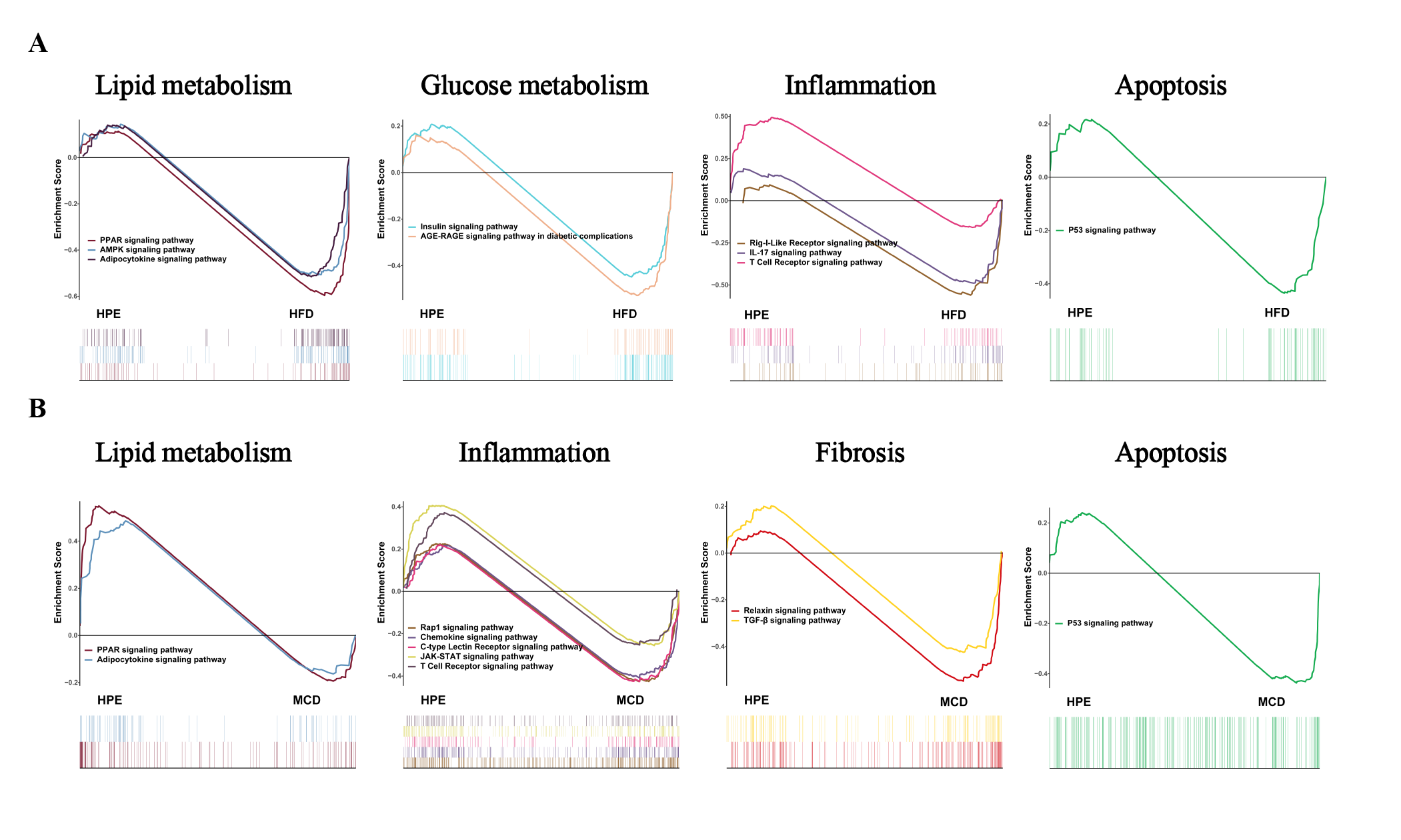

Supplement: Supplementary 1 — Figs. S1 to S8 Table S1 [file research.0276.f1.zip › Figure-S4.tif]

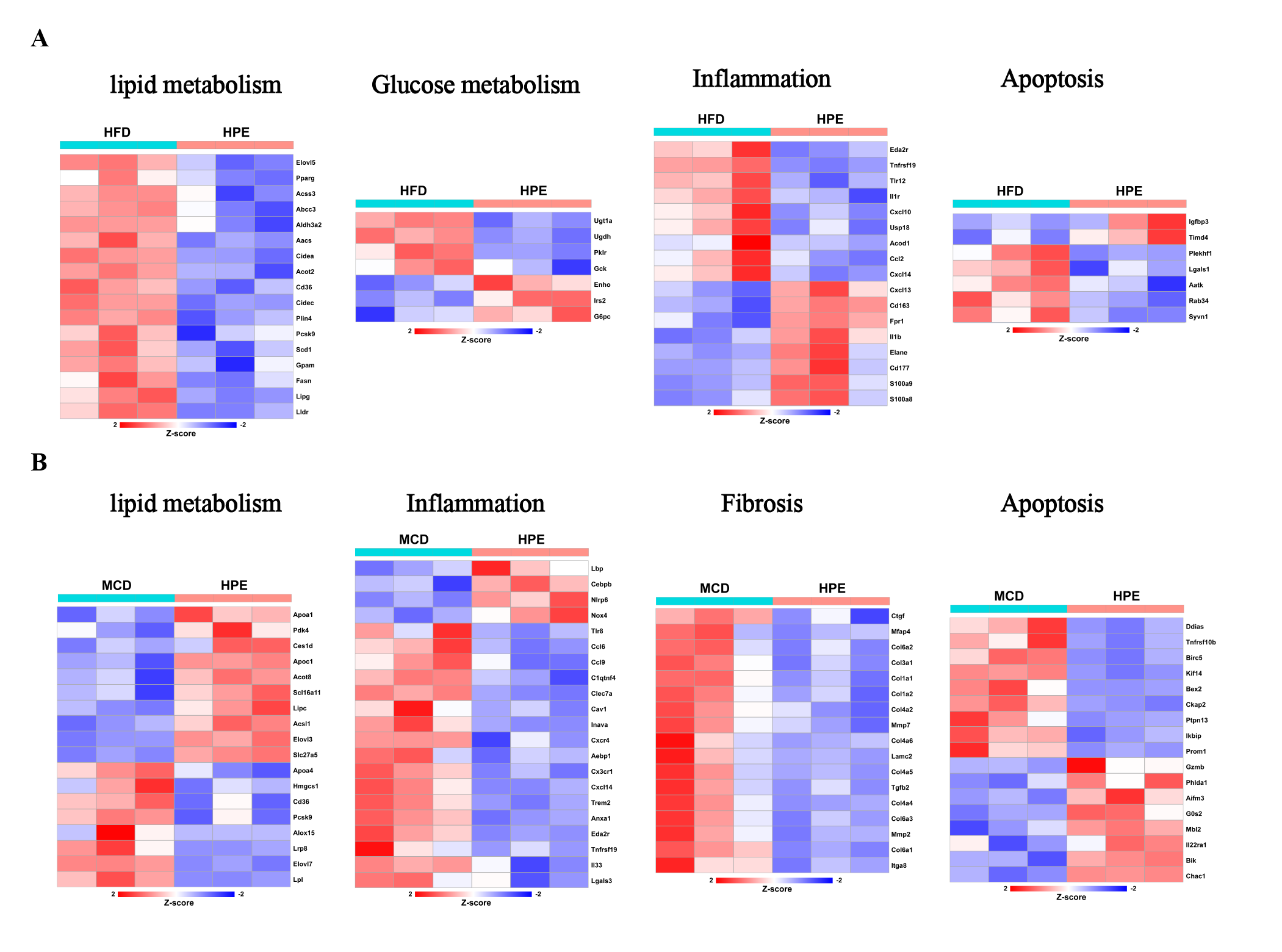

Supplement: Supplementary 1 — Figs. S1 to S8 Table S1 [file research.0276.f1.zip › Figure-S5.tif]

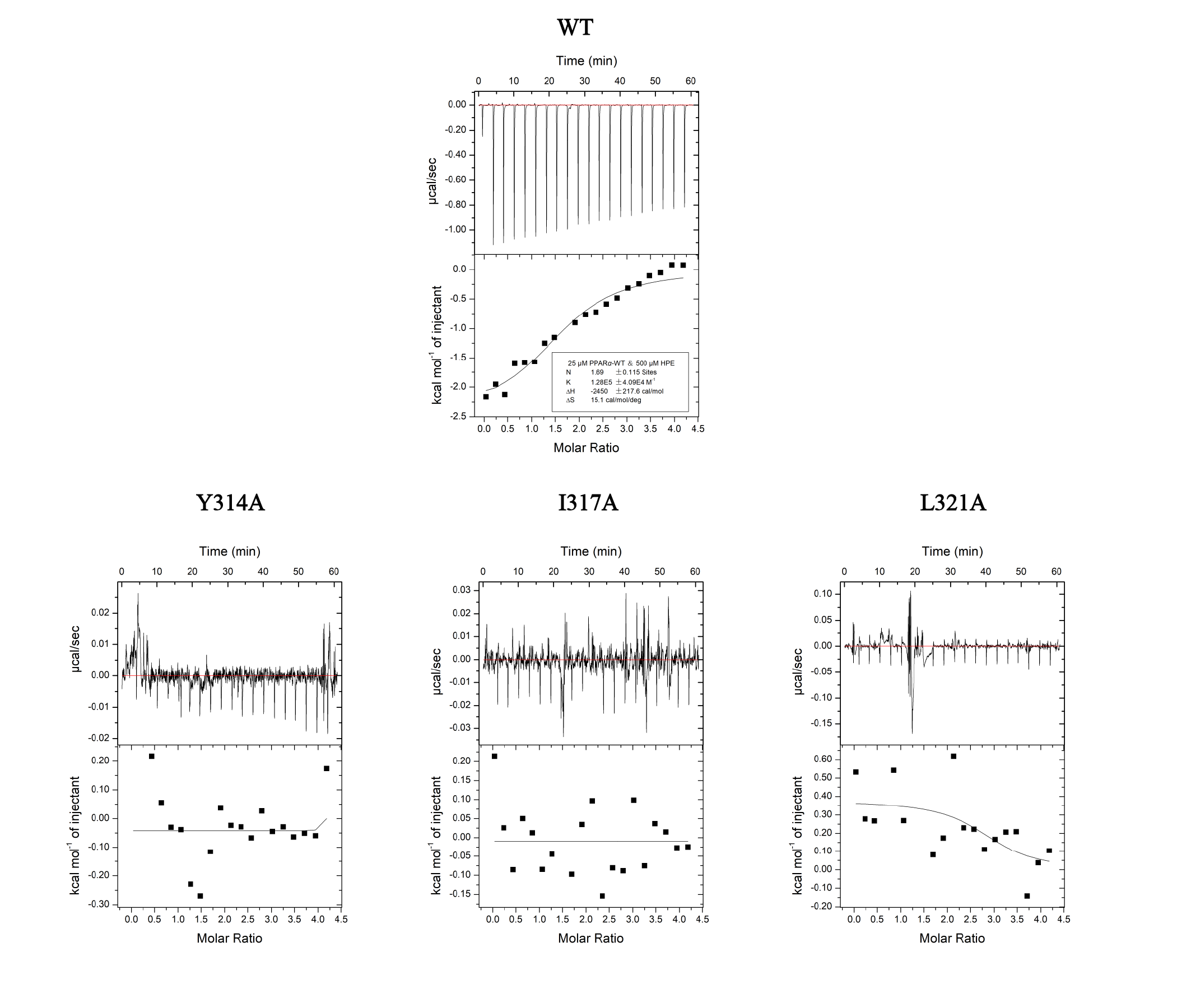

Supplement: Supplementary 1 — Figs. S1 to S8 Table S1 [file research.0276.f1.zip › Figure-S6.tif]

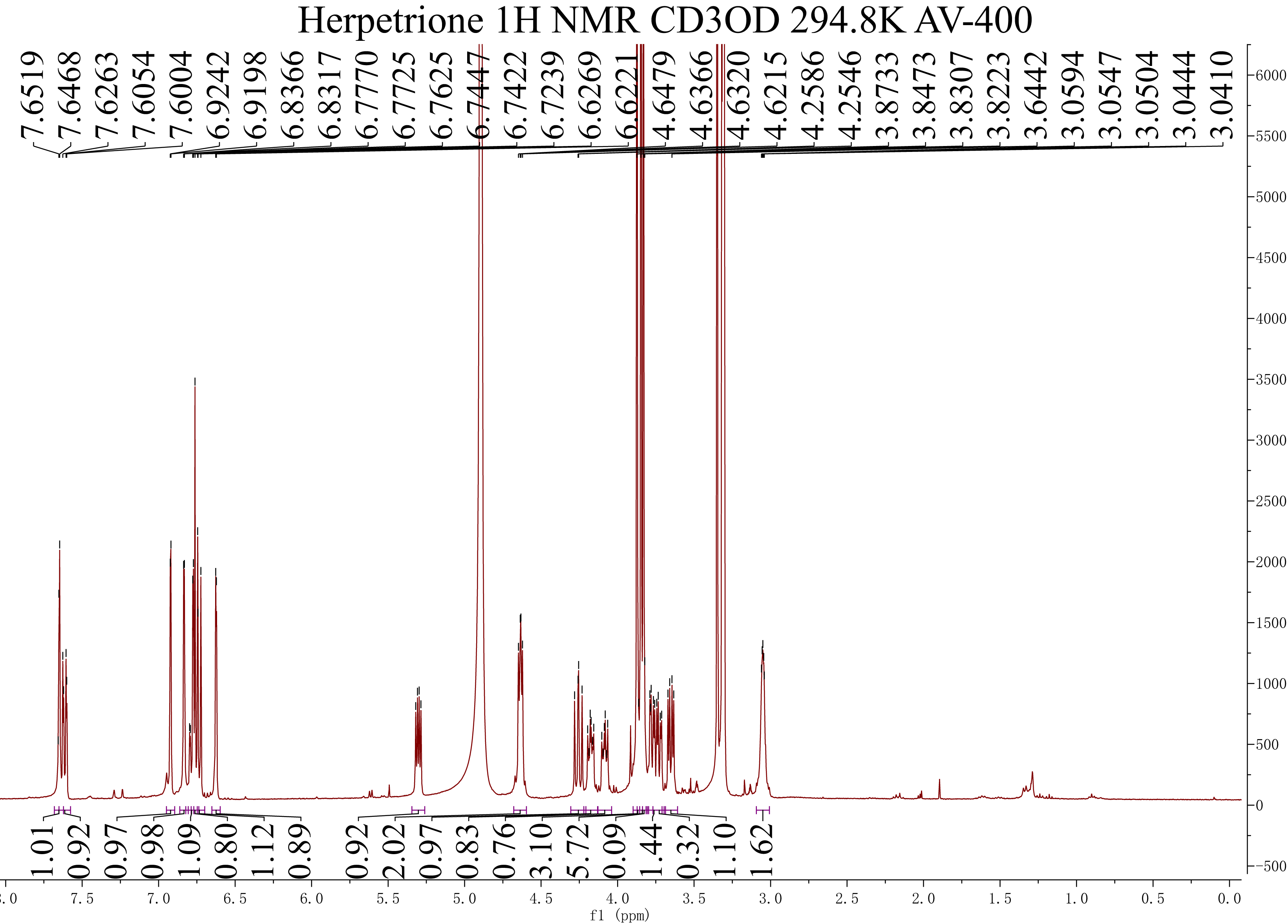

Supplement: Supplementary 1 — Figs. S1 to S8 Table S1 [file research.0276.f1.zip › Figure-S7.tif]

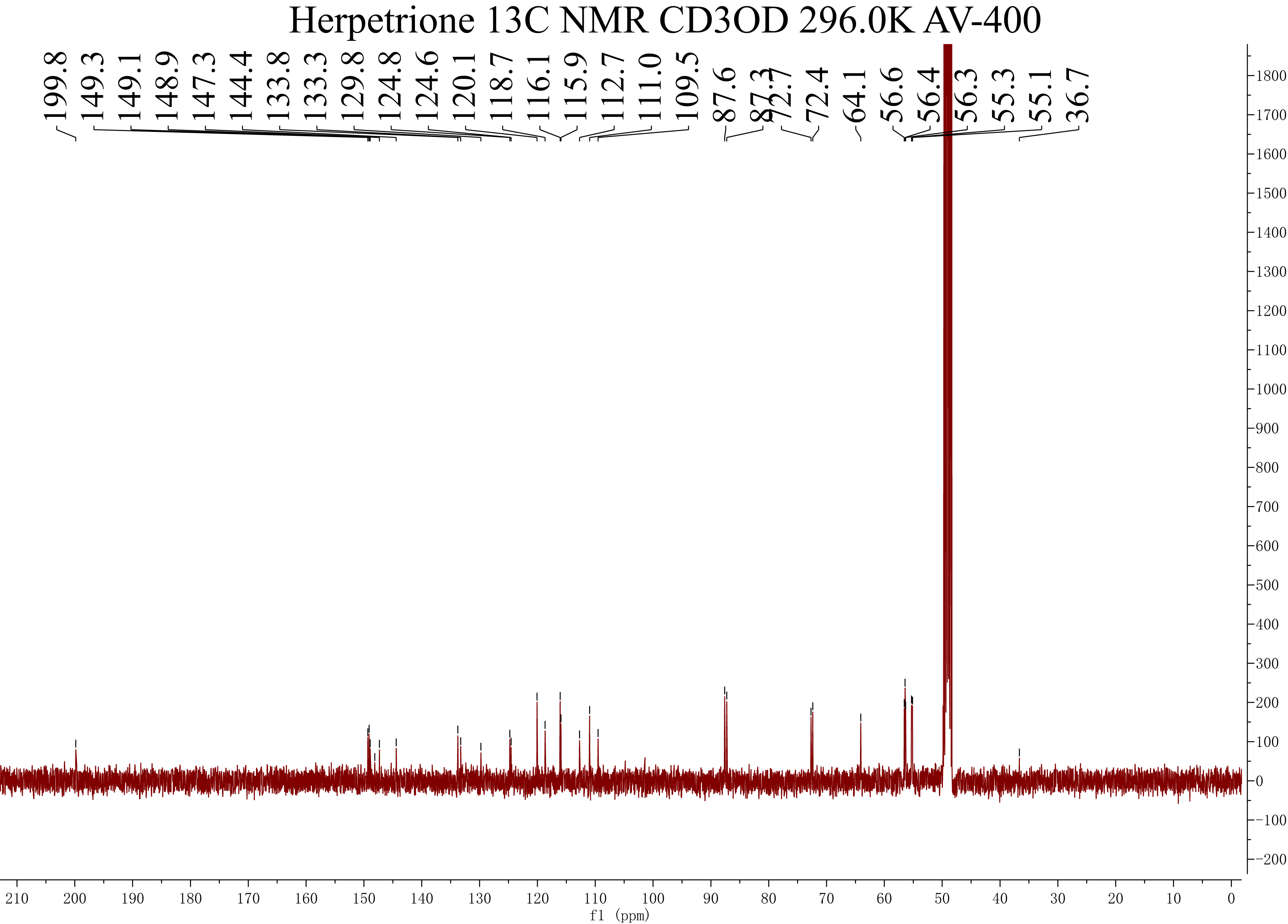

Supplement: Supplementary 1 — Figs. S1 to S8 Table S1 [file research.0276.f1.zip › Figure-S8.tif]
